# Molecular docking and QSAR studies for modeling the antimalarial activity of hybrids 4-anilinoquinoline-triazines derivatives with the wild-type and mutant receptor *pf*-DHFR

**DOI:** 10.1016/j.heliyon.2019.e02357

**Published:** 2019-08-26

**Authors:** Hanine Hadni, Menana Elhallaoui

**Affiliations:** Engineering Materials, Modeling and Environmental Laboratory, Faculty of Sciences Dhar El mahraz, Sidi Mohammed Ben Abdellah University, B.P. 1796, Atlas, Fes, Morocco

**Keywords:** Theoretical chemistry, Pharmaceutical chemistry, Molecular docking, Antimalarial activity, pf-DHFR, QSAR, 4-Anilinoquinoline-triazine

## Abstract

*Plasmodium falciparum dihydrofolate reductase (pf-DHFR)* is one of the several targets in the treatment of malaria. Double and quadruple mutations at residues 51, 59, 108, and 164 of *pf-DHFR* have been linked to antifolate resistance. Several efforts are underway to overcome this drug resistance and to produce potential inhibitors. In this regard, the quantitative structure-activity relationship (QSAR) and docking studies were performed for previously reported 4-anilinoquinoline and 1,3,5-triazines based molecular hybrids. The generated model showed good correlation coefficients (R^2^ = 0.70) and test set prediction coefficient (R^2^ = 0.74). These outcomes showed the good predictive competence of the established QSAR model. Based on these results we docked into active site of *pf-DHFR* protein with the most active (**4**) and the less active (**5**) compounds. The docking results revealed that these molecules interact specifically with SER108 and ILE164 in the *pf*-DHFR binding pocket as that of best active compound but also showed additional interactions with LEU40 and GLY44.

## 1 Introduction

Malaria is an infectious disease caused by parasites of the genus *Plasmodium* [1]. Five species of malaria parasites are known, namely, *P. falciparum*, *P. vivax*, *P. malariae*, *P. ovale* and *P. knowlesi* [2]. Approximately 90 % of deaths (generally of children in Africa) related to malaria infections are caused by *Plasmodium falciparum* [3, 4]. In 2016, 216 million of human beings are counted by the World Health Organization (WHO) over 91 countries of the world, which are affected by malaria, with an increase of 5 million cases compared to previous years [5].

**Fig. 1:**
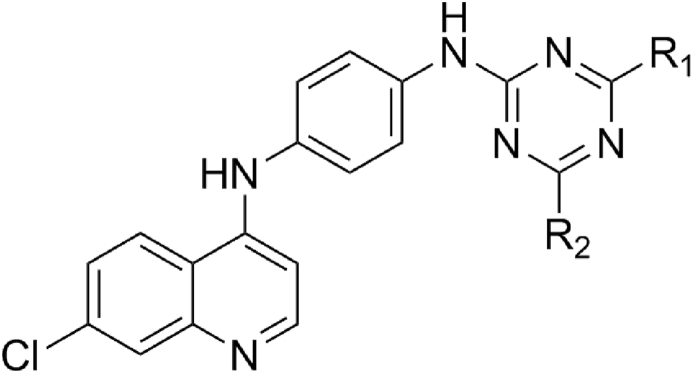
The structure of the 4-anilinoquinoline-triazine derivatives.

Despite of the great efforts devoted to discover an effective antimalarial drugs, these efforts suffer from many obstacles, including drug resistance issues [6, 7]. To overcome this problem, the concept of hybrid molecules has been introduced as one of the most used solutions, in which two or more pharmacophores are linked together and act by inhibiting simultaneously two conventional targets [8]. In this regard, the 1,3,5-triazine derivatives such as cycloguanil, chlorcycloguanil and WR99210are already approved as effective *dihydrofolatereductase* (*DHFR*) inhibitors, which selectively inhibit biochemical processes that are vital for parasite growth [9]. In addition, quinoline nucleus has attracted much interest of medicinal chemists, as an imperative pharmacophore accountable for imparting antimalarial action [10, 11]. Furthermore, to become a drug a novel synthetic molecule must take a very long journey. As a consequence, the pharmaceutical industry is moving towards new research methods, involving predicting the activities of molecules before they are even synthesized. The use of molecular modeling techniques such as QSAR and molecular docking has produced very impressive results in last year's [12, 13]. In the purpose to pursue our previous works [14, 15]. In this paper, we have performed the molecular modeling of 4-anilinoquinoline-triazines (Fig. 1) as a potential antimalarial compounds by using QSAR and docking studies [16].

Quantitative Structure Activity Relationships (QSAR) is very important tool in drug discovery [17]. The multidimensional molecular descriptors (1D, 2D, 3D) have been calculated to identify regions in space which are correlated to the biological activities (Table 1) [18]. QSAR models could be generated by using statistical methods. In this study, we have used a training set of 37 4-anilinoquinoline-triazines derivatives to build the QSAR model [19]. For this purpose, we used multiple linear regression analysis (MLR)) and artificial neural networks (ANN). The predictive ability of the established model has been tested by several validation techniques such as: internal and external validations as well as Y-randomization methods. *Plasmodium falciparum dihydrofolate reductase (pf-DHFR)* is the most important targets for antimalarial drug discovery. In addition, we have performed the docking molecular of two compounds, 4 and 5, with *Pf-DHFR* in its two forms; the wild type and the quadruple mutant [20]. Also we investigated the interaction of these hybrids with the binding site of *Pf-DHFR* protein structures, in order to gain structural insight for improved antimalarial activity.

**Table 1:** Studied compounds and their observed activities logIC_50_.

Compounds	R_1_	R_2_	IC_50_(obs)	LogIC_50_(obs)
1	3-fluoroanilinyl	4-methylpiperazin-1-yl	22.80	1.358
2	3-fluoroanilinyl	4-ethylpiperazin-1-yl	2.47	0.393
3	3-fluoroanilinyl	2-morpholinoethan-1-aminyl	40.02	1.602
4	3-fluoroanilinyl	3-morpholinopropan-1-aminyl	1.36	0.134
5	3-fluoroanilinyl	piperidin-1-yl	128.74	2.110
6	4-fluoroanilinyl	4-methylpiperazin-1-yl	8.97	0.953
7	4-fluoroanilinyl	4-ethylpiperazin-1-yl	11.11	1.046
8	4-fluoroanilinyl	2-morpholinoethan-1-aminyl	9.81	0.992
9	4-fluoroanilinyl	3-morpholinopropan-1-aminyl	15.82	1.199
10	4-fluoroanilinyl	piperidin-1-yl	41.75	1.621
11	3-chloroanilinyl	4-methylpiperazin-1-yl	2.41	0.382
12	3-chloroanilinyl	4-ethylpiperazin-1-yl	2.11	0.324
13	3-chloroanilinyl	2-morpholinoethan-1-aminyl	30.60	1.486
14	3-chloroanilinyl	3-morpholinopropan-1-aminyl	3.82	0.582
15	4-chloroanilinyl	4-methylpiperazin-1-yl	7.80	0.892
16	4-chloroanilinyl	4-ethylpiperazin-1-yl	4.14	0.617
17	4-chloroanilinyl	3-morpholinopropan-1-aminyl	4.63	0.666
18	3-methoxyanilinyl	4-methylpiperazin-1-yl	4.26	0.629
19	3-methoxyanilinyl	4-ethylpiperazin-1-yl	8.90	0.949
20	3-methoxyanilinyl	3-morpholinopropan-1-aminyl	2.98	0.474
21	4-methoxyanilinyl	4-ethylpiperazin-1-yl	4.26	0.629
22	4-methoxyanilinyl	3-morpholinopropan-1-aminyl	3.81	0.581
23	3,4-dimethoxyanilinyl	4-methylpiperazin-1-yl	5.04	0.702
24	3,4-dimethoxyanilinyl	4-ethylpiperazin-1-yl	2.58	0.412
25	3,4-dimethoxyanilinyl	3-morpholinopropan-1-aminyl	4.58	0.661
26	1,2,3,4-tetrahydroquinolinyl	4-methylpiperazin-1-yl	12.64	1.102
27	1,2,3,4-tetrahydroquinolinyl	4-ethylpiperazin-1-yl	20.84	1.319
28	1,2,3,4-tetrahydroquinolinyl	2-morpholinoethan-1-aminyl	17.35	1.239
29	1,2,3,4-tetrahydroquinolinyl	3-morpholinopropan-1-aminyl	14.70	1.167
30	1,2,3,4-tetrahydroisoquinolin-2(1H)-yl	4-methylpiperazin-1-yl	28.12	1.449
31	1,2,3,4-tetrahydroisoquinolin-2(1H)-yl	4-ethylpiperazin-1-yl	12.12	1.084
32	1,2,3,4-tetrahydroisoquinolin-2(1H)-yl	2-morpholinoethan-1-aminyl	45.21	1.655
33	1,2,3,4-tetrahydroisoquinolin-2(1H)-yl	3-morpholinopropan-1-aminyl	20.32	1.308
34	morpholinyl	4-methylpiperazin-1-yl	3.22	0.508
35	morpholinyl	4-ethylpiperazin-1-yl	2.16	0.334
36	morpholinyl	2-morpholinoethan-1-aminyl	5.87	0.769
37	morpholinyl	3-morpholinopropan-1-aminyl	2.76	0.441

## 2 Materials and methods

### 2.1 Studied molecules

To perform the molecular modeling we have taken the experimental antimalarial activities data of 37 hybrids molecules described previously [16]. Thus, the observed activities (IC50) are converted into logarithm scale logIC50 and they are presented in Table 1.

### 2.2 Molecular descriptors calculation

In order to build a reliable QSAR model, a total of 14 descriptors including lipophilic, geometrical, physicochemical, and steric. Those are calculated with the MM2 method using ACD/ChemSketch [21] and ChemBioOffice softwares [22]. Meanwhile, the geometry of the studied compounds was optimized using DFT/B3LYP(6-31G) method [23, 24]. The electronic descriptors were calculated by Gaussian 03 quantum chemistry software [25]. All descriptors used in this work are presented in Table 2.

**Table 2:** List of the calculate descriptors.

Type of descriptors	Electronic	lipophilic	Geometrical	physicochemical	Steric
Name of the descriptors	HOMO energy (E_HOMO_) LUMO energy (E_LUMO_) Dipole moment (Dp) total energy (E)	lipophilic (Octanol-water partition coefficient) (LogP) VDW energy (E_VDW_)	Torsion energy (T) stretch-bend energy (SB)	Critical pressure (CP) Critical volume (CV)	Density (D) Refractive Index (R) Surface Tension (ST) parchor (P)

### 2.3 Statistical methods

In the aim to build QSAR model, we have chosen a set of 37 compounds from previously reported work, whose shown important antimalarial activity [16]. The complete set was randomly divided into two subsets a training set (29 compounds) to build the model and a test set (8 compounds) to evaluate the reliability of the established model. Various statistical methods were used to build the QSAR model, viz: Multiple Linear Regression (MLR) [26], and Artificial Neural Networks (ANN) [27]. Indeed, the MLR with descendent selection of variables was used to study the relation between one dependent variable (antimalarial activity) and several independent variables (calculated molecular descriptors). Furthermore, the MLR was used to select the descriptors that will serve as the input parameters for ANN. Hence, ANN could be considered as suitable tools that have powerful mechanism to capture patterns in data, which has been widely used to model nonlinear system [28]. In addition, the high values of the correlation coefficient indicate how the equations fit the data. On other hand, validation a strategy has been recognized to inquire into the applicability of the QSAR models on a new data. For this reason, we have used Cross-Validation with “leave-one-out” procedure, in order to explore the reliability of the proposed models. Cross validation was used in which a number of models were developed with one sample ignored each time. The model was evaluated by measuring its accuracy in predicting the responses of the remaining data (the ones that have not been used in the development of the model) [29, 30].

According to Golbraikh and Tropsha study on validation methods, cross-validation is necessary but not sufficient to ensure the predictive capability of the proposed QSAR model. In this study, we have validated the QSAR model by both MLR and ANN methods based on training set. The external validation should be in perfect agreement with the criteria of Golbraikh and Tropsha [31]. Finally, Y-randomization test has been used to exclude the possibility of random correlation between descriptors and its corresponding bioactivities in the obtained model. This test consists to mix randomly many properties/experimental activities for the new learning series using the same descriptors. For an acceptable QSAR model, the average correlation coefficient (Rr) of randomized models should be less than the correlation coefficient (R) of nonrandomized model [32].

### 2.4 Molecular docking modeling

A molecular modeling study was conducted in order to gain insight into the key structural requirements of a geometrical model and to analyze the interactions of the hybrid systems with the active sites of the protein *pf-DHFR* [14, 20] of both wild (coded as 1J3I.pdb) and quadruple mutant types (coded as 1J3K.pdb), which are obtained from the Protein Data Bank RCSB [33]. In this study, we have performed the molecular docking of two compounds with *pf-DHFR* protein. We have chosen the highest active compound (compound number 4) and the lowest active compound (compound number 5) of the studied series. First, we have removed all water molecules from the receptor, the ligands and non-protein parts by using the Discovery Studio software [34]. The AutoDock 4.2, has been used to analyze the interactions between the ligand and the protein [35]. The 3D grid was created by the AUTOGRID algorithm [36]to evaluate the interacting energy between ligands and wild-type protein. The grid maps were constructed using 60, 60 and 60 pointing in x, y and z directions, with grid point spacing of 0.375° A. The center grid box is about (30.323Å, 5.116Å and 58.385Å) by the ligand location in the complex. However, the center grid box of the quadruple mutant type protein is about (29.987Å, 5.56 Å and 57.424 Å). Discovery Studio software was used for the 2D and 3D visualizations of the established interactions [34].

## 3 Results and discussion

To perform this study, we have divided the 37 studied compounds randomly into training sets and test sets which containing 29 and 8 compounds respectively. The values of the selected descriptors and the predicted values of antimalarial activity of the training set which obtained by using MLR, ANN and CV methods are presented in Table 3.

**Table 3:** The values of selected descriptors and observed/predicted activity (logIC_50_).

N		E	S–B	T	E_VDW_	LogP	ST	LogIC_50_ (Obs)	MLR	ANN	Cv(LOO)
2		-2213.007	0.520	-15.547	42.004	7.347	72.100	0.393	0.7412	0.724	0.739
3		-2288.181	0.532	-12.073	43.096	6.065	77.900	1.602	1.1958	1.311	1.299
4		-2327.486	0.556	-12.039	43.729	6.170	75.700	0.134	0.8582	0.667	0.772
5		-2118.390	0.371	-15.879	39.069	7.986	76.600	2.110	2.1119	2.099	2.100
7		-2213.006	0.522	-15.540	42.003	7.347	72.100	1.046	0.7385	0.725	0.737
8		-2288.181	0.534	-12.048	43.091	6.065	77.900	0.992	1.1959	1.310	1.298
9		-2327.485	0.558	-12.034	43.726	6.170	75.700	1.199	0.8560	0.665	0.769
11		-2534.064	0.483	-15.753	41.898	7.409	76.700	0.382	0.7792	0.649	0.628
12		-2573.369	0.533	-15.553	42.645	7.747	73.800	0.324	0.5348	0.473	0.472
13		-2648.544	0.545	-12.055	43.739	6.465	79.600	1.486	0.9925	1.416	1.288
14		-2687.848	0.570	-12.044	44.369	6.570	77.400	0.582	0.6502	0.629	0.740
15		-2534.064	0.485	-15.692	41.838	7.409	76.700	0.892	0.7776	0.641	0.625
16		-2573.369	0.533	-15.557	42.558	7.747	73.800	0.617	0.5202	0.461	0.464
18		-2188.972	0.518	-15.798	44.081	6.724	73.200	0.629	0.7116	0.675	0.678
19		-2228.277	0.571	-15.560	44.843	7.062	70.600	0.949	0.5258	0.762	0.761
20		-2342.756	0.624	-12.094	46.444	5.886	74.100	0.474	0.5699	0.495	0.515
21		-2228.275	0.578	-15.558	44.824	7.062	70.600	0.629	0.5114	0.764	0.756
22		-2342.754	0.615	-12.040	46.569	5.886	74.100	0.581	0.6138	0.527	0.550
23		-2303.447	0.477	-13.677	47.151	6.598	70.600	0.702	0.7632	0.718	0.768
25		-2457.232	0.593	-9.705	49.569	5.759	71.600	0.661	0.6775	0.659	0.611
26		-2191.186	0.989	-15.240	49.085	7.482	71.700	1.102	1.2207	1.110	1.177
27		-2230.491	1.046	-14.930	49.892	7.820	69.200	1.319	1.0648	1.320	1.174
28		-2305.667	1.058	-11.375	51.018	6.538	74.400	1.239	1.4323	1.243	1.280
30		-2191.189	0.605	-15.188	46.528	7.381	71.700	1.449	1.3842	1.403	1.300
31		-2230.494	0.660	-14.869	47.331	7.719	69.200	1.084	1.2315	1.108	1.204
32		-2305.670	0.669	-11.361	48.401	6.437	74.400	1.655	1.5870	1.661	1.600
34		-2074.665	0.796	-6.763	43.187	5.271	72.000	0.508	0.5254	0.508	0.392
35		-2113.970	0.851	-6.497	43.975	5.609	69.300	0.334	0.3270	0.356	0.390
37		-2228.449	0.885	-3.101	45.637	4.433	72.900	0.441	0.4175	0.436	0.427

### 3.1 Multiple linear regression

**Fig. 2:**
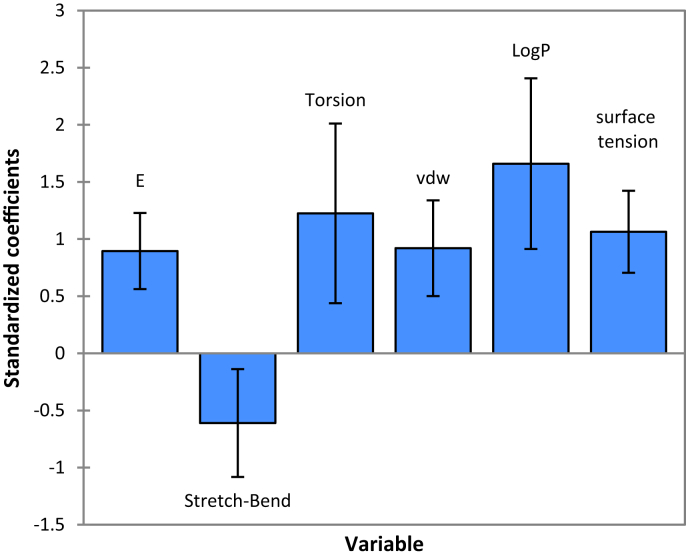
Modeling characterization by the normalized coefficients.

**Fig. 3:**
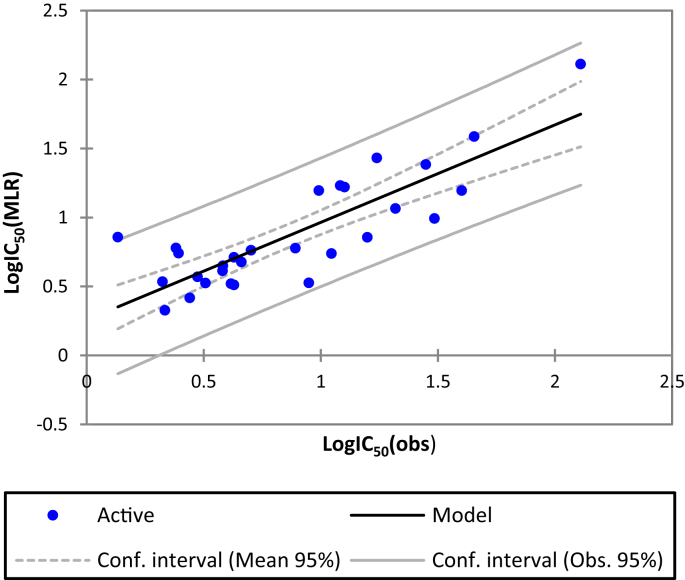
The correlation between the observed and the predicted activities.

The MLR method is based on three criteria: Coefficient of determination (R^2^), the root mean square error (RMSE) and the Fisher ratio value (F) [14]. The MLR results which contain the corresponding normalized descriptors coefficients and the correlation between the observed and predicted activities are presented in Figs. 2 and 3 respectively. In addition, the QSAR model of the training set built is represented by the following Eq. (1):

(1) LogIC_50_ = -15.8 + 0.0026*E – 1.67* S–B + 0.18*T + 0.15*VDW + 0.91 LogP + 0.18* ST

N = 29 R = 0.84 R^2^ = 0.70 F = 8.84 RMSE = 0.29

The selected descriptors involved in the MLR model of the training set are: Total energy (E), stretch-bend energy (SB), Torsion energy (T), VDW energy (E_VDW_), lipophilic (LogP) and Surface Tension (ST). According to the coefficient normalization diagram, we have found that the built model presented four most important descriptors (LogP, T, ST and S-b) correlated with Log IC50 with high value of R^2^ (0.7). Indeed, the most important parameter in the model is LogP (coefficient of 0.91). The positive sign of the Log P Eq. (1), indicates that the larger the Log P value, the lower the activity of the compound is. From Table 3, compounds 4 and 5, which have respectively the lowest and the largest Log P values, also have the greatest and the minor antimalarial activities. The compound 4 possess large alkyl chains; therefore, it is possible that there are hydrophobic interactions between the substituent and the receptor.

The second most important descriptor in Model 1 is the T and ST (coefficient of 0.18). The number of torsion plays a very important role in the activity, this is due to the flexibility of the molecule inside the active site. Moreover, the Surface tension is closely related to the forces of intermolecular attraction. The stronger the intermolecular forces are, the more tightly the molecules are held together in the liquid phase and, therefore the higher the surface tension will be. T and ST appears in the Eq. (1) with apositive sign which shows that the molecules with higher value of T and ST have the lowest antimalarial activity.

The third most important descriptor in model 1 is S–B (coefficient of -1.67). The S–B a geometry parameter, deals with the stretching and bending or one can say the conformational flexibility of the molecule. The descriptor S–B exhibits negative correlation with LogIC50, so the substituents that increase the stretch-bend energy of the compound will also enhance the antimalarial activity.

High correlation coefficient (R = 0.84) of the built QSAR model based on the training set indicates good variance explanation of the model, further supported by low standard deviation (RMSE = 0.23). Furthermore, evaluation of the degree of visual significance of the Fischer Test (F) confidence (p < 0.0001) reflects the good predictive competence of the generated model.

### 3.2 Artificial neural networks

**Fig. 4:**
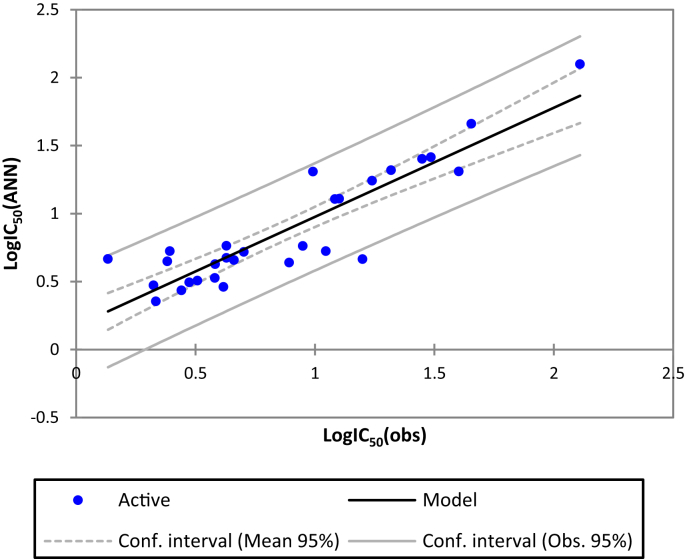
The correlation between the observed and the predicted activities established by ANN.

In this study, we have used 3 layers Neural Networks: The input layer that contains six neurons representing the selected descriptors, the output layer which represent the observed activity values (logIC_50_) and the hidden layer. It should be noted that there are no theoretical or empirical rules to determinate the number of hidden layers. While, few authors [37, 38] recommended to take into consideration *ρ*= (number of weight)/(number of connection) parameter which must in the range of 1 < ρ < 3 [39,40]. Thus, the final ANN architecture is (6-2-1). The Fig. 4 shows the correlation between the observed and the predicted activities established by the ANN.

The plot of observed versus predicted activity (Fig. 4) shows a good fit with R^2^ value of 0.81 and the mean squared error RMSE = 0.18 indicate that model possesses a significant statistical quality and the selected descriptors by MLR are pertinent.

### 3.3 Cross Validation

**Fig. 5:**
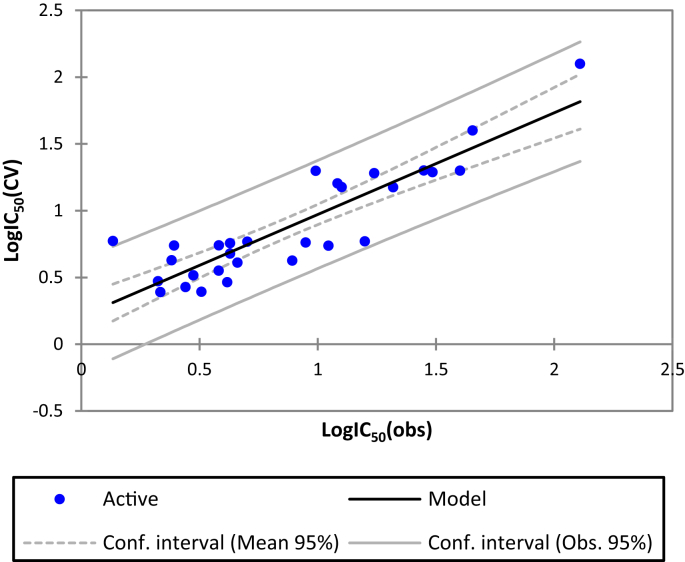
Correlation of observed and predicted activities calculated using Cross-Validation (LOO).

The results obtained by Cross Validation (CV)with “leave-one-out”are represented in Fig. 5. Therefore, the obtained parameters R^2^ = 0.78 and RMSE = 0.19 shows that the built QSAR model is not sensitive to the CV. Which obviously, indicate that the proposed QSAR model is stable and robust. However, Cross Validation is not a good parameter to estimate the ability of QSAR models according to Golbraikh and Tropsha study [31].

### 3.4 Y-randomization

Herein, we have randomly mixed descriptors and observed activity of the newly training set which contains 29 compounds obtained by excluding 8 compounds and adding 8 compounds of test set. Then, we build new model through MLR methods us described previously. The obtained results are given in Table 4.

**Table 4:** Calculated results using Y-randomization.

N	4	23	5	26	30	37	2	32	24	21	28	9	7	20	11
logIC_50_	0.134	0.702	2.110	1.102	1.449	0.441	0.393	1.655	0.412	0.629	1.239	1.199	1.046	0.474	0.382
pred logIC_50_	0.869	0.732	2.102	1.254	1.368	0.401	0.774	1.534	0.588	0.543	1.428	0.867	0.772	0.585	0.802
N	36	1	27	25	16	19	13	31	8	12	34	17	33	15	-
logIC_50_	0.769	1.358	1.319	0.661	0.617	0.949	1.486	1.084	0.992	0.324	0.508	0.666	1.308	0.892	-
pred logIC_50_	0.716	1.032	1.080	0.610	0.528	0.556	0.980	1.196	1.211	0.541	0.545	0.626	1.261	0.800	-

The newly QSAR model using the Y-randomization method is represented by the following Eq. (2):

(2) LogIC50 = -15.08 + 0.0026*E - 1.44*S–B +0.16*T + 00.14*VDW + 0.85*LogP + 0.17*ST

N = 29 R = 0.83 R^2^ = 0.69 F = 8.14 RMSE = 0.29

The obtained correlation coefficient value R^2^ = 0.69 of the newly training set is compared to the obtained using the first training set. The results of Y-randomization confirm the absence of dependence between descriptors included in the QSAR model.

### 3.5 External validation

The study conducted by Golbraikh and Tropsha on validation methods lead to the insufficient of the internal validation methods to confirm the reliability of the built QSAR models. For this reason, external validations are hardly needed to build a reliable QSAR model [31]. The later must respect some important criteria recommended by Golbraikh and Tropsha. In Table 5, we present the test set containing 8 compounds that has been reserved to external validation, maintaining their original numbers and taken from Table 3 with their observed and predicted activity by MLR and ANN models. The results of Golbraikh and Tropsha criteria's validation are presented in Table 6 [31].

**Table 5:** The results of external validation by the MLR and ANN methods.

N	LogIC_50_	Pred (LogIC_50_) MLR	Residual	Pred (LogIC50)	Residual
1	1.358	0.99	0.388	0.776	0.443
6	0.953	0.984	-0.013	0.769	0.042
10	1.621	2.119	-0.158	2.099	-0.109
17	0.666	0.65	-0.061	0.611	-0.146
24	0.412	0.646	-0.312	0.525	-0.347
29	1.167	1.155	0.079	1.014	0.106
33	1.308	1.326	0.097	1.393	0.013
36	0.769	0.736	-0.02	0.544	-0.002

**Table 6:** Golbraikh and Tropsha criteria.

Parameter	Formula	Threshold	Modelscore
$R_{ANN\;ext}^{2}$	$R_{ext}^{2}=1-\frac{\sum {\left(Y_{pred\left(test\right)}-Y_{\left(test\right)}\right)}^{2}}{\sum {\left(Y_{\left(test\right)}-{\bar{Y}}_{tr}\right)}^{2}}$	$R_{ext}^{2}$>0.6	0.68
r^2^	Coefficient of determination for the plot of predicted versus observed for test set by MLR	r^2^ > 0.6	0.74
$r_{0}^{2}$	r^2^ at zero intercept		0.67
$r_{0}^{'2}$	r2 for the plot of observed versus predicted activity for the test set at zero intercept		0.68
$\left|r_{0}^{2}-r_{0}^{'2}\right|$		$\left|r_{0}^{2}-r_{0}^{'2}\right|<0.3$	0.01
k	Slope of the plot of predicted versus observed activity for test set at zero intercept	0.85 < k < 1.15	0.92
$\frac{r^{2}-r_{0}^{2}}{r^{2}}$		$\frac{r^{2}-r_{0}^{2}}{r^{2}}<0.1$	0.07
k^’^	Slope of the plot of observed versus predicted activity at zero intercept	0.85 < k^’^<1.15	0.93
$\frac{r^{2}-r_{0}^{'2}}{r^{2}}$		$\frac{r^{2}-r_{0}^{'2}}{r^{2}}<0.1$	0.08

Overall, we can conclude that Golbraikh and Tropsha criteria's and external validation are successfully validated, which indicate that the built QSAR model is in perfect agreement with all validation methods in one hand. In the other hand, the experimental antimalarial activity could be accurately predicted using the established QSAR model.

### 3.6 Molecular docking study

**Fig. 6:**
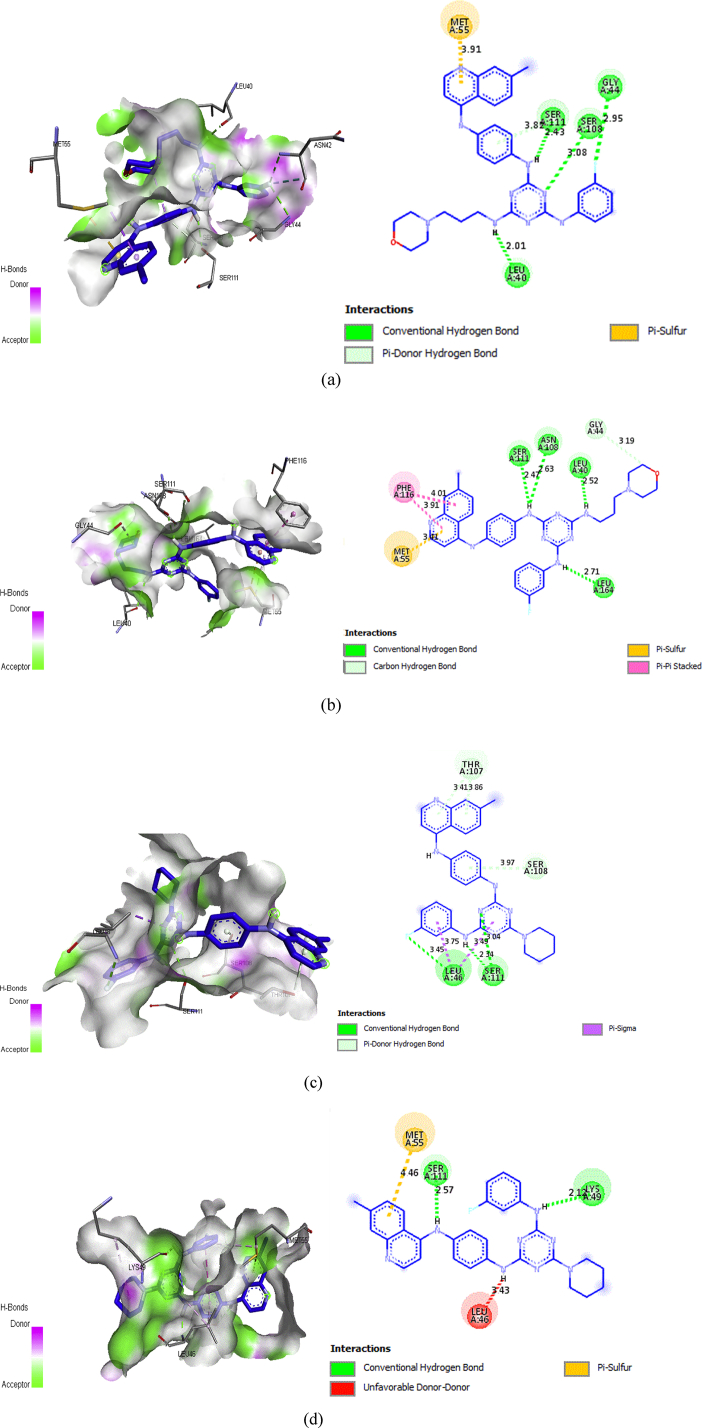
2D and 3D docking poses showing interactions of compounds 4 and5 in the binding sites of wild type and quadruple mutant of pf-DHFR-TS. (a) Compound 4: wild type of *pf-DHFR* (binding energy −10.6 kcal/mol). (b) Compound 4: quadruple mutant of pf- DHFR-TS (binding energy −10.9 kcal/mol). (c) Compound 5: wild type of *pf-DHFR* (binding energy −11.4 kcal/mol). (d) Compound 5: quadruple mutant of pf-DHFR-TS (binding energy −10.3 kcal/mol).

The molecular Docking study was performed for two reasons. The first is to understand the good antimalarial activity potency manifested with some compounds. The second is to find out the key interaction types established with the protein *(pf-DHFR)* in its two type (wild and mutant) [14]. The reported study of Yuvanyama et al [41] has found the binding modes, and has localized the active sites in wild and mutant of protein *(pf-DHFR).* The study performed with a potent inhibitor 1,3,5-triazine derivative which is a preclinical molecule called WR99210. It is found that the important sites in the case of the wild type are located in Ile14, Ala16, Met55, Asp54, Ser108, Ile164 and Tyr170. It was also found that important sites are located in Ala16, Cys50, Asn51, Cys59, Asn108, Leu164 and Tyr170 in the case of the wild type. The interactions mode obtained by molecular docking for compounds 4 and 5 are presented in Fig. 6.

In the case of the wild type, compound 4 performs four interactions through hydrogen bonding with SER111, LEU40, SER108 and GLY44 amino acids. The interactions involve three nitrogen atoms linked to the triazine system and one fluorine atom linked to benzene group, with the distance of 2.43 Ǻ, 2.01 Ǻ, 3.08 Ǻ and 2.95 Ǻ, respectively. However, compound 5 performs only three hydrogen bonds with less important binding sites, Ser111 and Gly46, as they are not cited as active sites for antimalarial activity. In the case of quadruple mutant, compound 4performs four hydrogen bonds between four nitrogenatoms linked to the triazine group and SER111, ASN108, LEU164 and LEU40 amino acids with the distance of 2.47 Ǻ, 2.63 Ǻ, 2.71 Ǻ and 2.52 Ǻ, respectively. For compound 5 showed only two hydrogen bonding interaction with less important binding sites SER 111 and LYS49.

In summary, the interactions formed by the compounds 4 with the binding sites of *pf-DHFR* are in good agreement with the previous study for antimalarial activity [41]. Moreover, no significant interactions with critical amino acid in *pf-DHFR* protein on both wild and mutant types, showed for compound 5. When, we have noticed for compound 4 bearing N-methy-3-morpholinopropan-1-amine substitute, the number of rotations increased 11 rotations versus 7 rotations in the case of compound 5. This could make molecule more flexible inside the active site of the protein. Furthermore, the introduction of new nitrogen atoms in the compound 4 influence on the number of hydrogen bond compared to compound 5. These outcomes, can explain the difference of measured activity between compound 4 and compound 5.

## 4 Conclusion

In the purpose of producing new effective antimalarial drugs, a QSAR model was developed using the in vitro antimalarial data reported. The built model was statistically significant and the significance was validated. Molecular docking study highlight the exclusive binding signature of the ligands with the active site residue i.e. ILE164, SER108 and LEU40of the target and it explains the specificity and subtle differences in their predicted IC50 values. The study has provided insights to improve biological activity with the change of 1-methylpiperidine by 3-morpholinopropan-1-aminein 4-aminoquinoline-triazinederivatives.

## Declarations

### Author contribution statement

Hanine Hadni: Conceived and designed the experiments; Analyzed and interpreted the data; Wrote the paper.

Menana Elhallaoui: Conceived and designed the experiments; Contributed reagents, materials, analysis tools or data.

### Funding statement

This research did not receive any specific grant from funding agencies in the public, commercial, or not-for-profit sectors.

### Competing interest statement

The authors declare no conflict of interest.

### Additional information

No additional information is available for this paper.
